# A microbiological and genomic perspective of globally collected *Escherichia coli* from adults hospitalized with invasive *E. coli* disease

**DOI:** 10.1093/jac/dkae182

**Published:** 2024-07-13

**Authors:** Enya Arconada Nuin, Tuba Vilken, Basil Britto Xavier, Joachim Doua, Brian Morrow, Jeroen Geurtsen, Oscar Go, Bart Spiessens, Michal Sarnecki, Jan Poolman, Marc Bonten, Miquel Ekkelenkamp, Christine Lammens, Herman Goossens, Youri Glupczynski, Sandra Van Puyvelde, COMBACTE-NET Consortium/EXPECT Study Group, Gert Leten, Sofie Van Mieghem, Madison Violette, Sonal Munshi, Moussa Aitabi, Anna Maria Azzini, Elda Righi, Nicola Duccio Salerno, Giuliana Lo Cascio, Eleonora Cremonini, Álvaro Pascual, Reinaldo Espíndola, Virginia Palomo, Olivier Barraud, Sarah V Walker, Naomi Akai, Risa Kimura, Louis Lakatos, Killian De Blacam, Joshua Thaden, Felicia Ruffin, Michael Dagher

**Affiliations:** Laboratory of Medical Microbiology, Vaccine & Infectious Disease Institute, University of Antwerp, Antwerp, Belgium; Laboratory of Medical Microbiology, Vaccine & Infectious Disease Institute, University of Antwerp, Antwerp, Belgium; Laboratory of Medical Microbiology, Vaccine & Infectious Disease Institute, University of Antwerp, Antwerp, Belgium; Department of Medical Microbiology and Infection Control, University of Groningen, University Medical Center Groningen, Groningen, The Netherlands; Janssen Research & Development, Infectious Diseases & Vaccines, Janssen Pharmaceutica, Beerse, Belgium; Janssen Research & Development, Raritan, NJ, USA; Bacterial Vaccines Discovery & Early Development, Janssen Vaccines & Prevention B.V., Leiden, The Netherlands; Janssen Research & Development, Raritan, NJ, USA; Janssen Research & Development, Infectious Diseases & Vaccines, Janssen Pharmaceutica, Beerse, Belgium; Janssen Vaccines, Branch of Cilag GmbH International, Bern, Switzerland; Bacterial Vaccines Discovery & Early Development, Janssen Vaccines & Prevention B.V., Leiden, The Netherlands; Julius Center for Health Sciences and Primary Care, University Medical Center Utrecht, Utrecht University, Utrecht, The Netherlands; ECRAID, Utrecht, The Netherlands; Department of Medical Microbiology, University Medical Center Utrecht, Utrecht, The Netherlands; Laboratory of Medical Microbiology, Vaccine & Infectious Disease Institute, University of Antwerp, Antwerp, Belgium; Laboratory of Medical Microbiology, Vaccine & Infectious Disease Institute, University of Antwerp, Antwerp, Belgium; Laboratory of Medical Microbiology, Vaccine & Infectious Disease Institute, University of Antwerp, Antwerp, Belgium; Laboratory of Medical Microbiology, Vaccine & Infectious Disease Institute, University of Antwerp, Antwerp, Belgium; Cambridge Institute of Therapeutic Immunology and Infectious Disease, University of Cambridge School of Clinical Medicine, Cambridge Biomedical Campus, Cambridge CB2 0AW, UK

## Abstract

**Objectives:**

*Escherichia coli* can cause infections in the urinary tract and in normally sterile body sites leading to invasive *E. coli* disease (IED), including bacteraemia and sepsis, with older populations at increased risk. We aimed to estimate the theoretical coverage rate by the ExPEC4V and 9V vaccine candidates. In addition, we aimed at better understanding the diversity of *E. coli* isolates, including their genetic and phenotypic antimicrobial resistance (AMR), sequence types (STs), O-serotypes and the bacterial population structure.

**Methods:**

Blood and urine culture *E. coli* isolates (*n* = 304) were collected from hospitalized patients ≥60 years (*n* = 238) with IED during a multicentric, observational study across three continents. All isolates were tested for antimicrobial susceptibility, O-serotyped, whole-genome sequenced and bioinformatically analysed.

**Results:**

A large diversity of STs and of O-serotypes were identified across all centres, with O25b-ST131, O6-ST73 and O1-ST95 being the most prevalent types. A total of 45.4% and 64.7% of all isolates were found to have an O-serotype covered by the ExPEC4V and ExPEC9V vaccine candidates, respectively. The overall frequency of MDR was 37.4% and ST131 was predominant among MDR isolates. Low in-patient genetic variability was observed in cases where multiple isolates were collected from the same patient.

**Conclusions:**

Our results highlight the predominance of MDR O25b-ST131 *E. coli* isolates across diverse geographic areas. These findings provide further baseline data on the theoretical coverage of novel vaccines targeting *E. coli* associated with IED in older adults and their associated AMR levels.

## Introduction

*Escherichia coli* is the most common cause of Gram-negative infections in humans, possessing the ability to infect extra-intestinal sites such as the urinary tract as well as normally sterile body sites resulting in invasive *E. coli* disease (IED) that includes acute infections such as bacteraemia, severe pyelonephritis and sepsis. Although IED affects all age categories, the incidence increases by age with adults aged 60 years or older having an increased risk of developing the disease. Case-fatality rates range from 13% to 19% but may reach up to 60% in elderly with healthcare-associated infections.^1–3^ The increase in antimicrobial resistance (AMR) among *E. coli* strains, such as *E. coli* O-serotype O25b:H4-ST131 represents a major challenge for the treatment and management of *E. coli* infections.^4^ Global morbidity and hospitalization due to IED are substantial and are expected to further increase driven by ageing populations and high overall AMR prevalence.^1^

The O-antigen, a component of the surface polysaccharide is the target of the serum’s opsonophagocytic killing activity that elicits an immunogenic response in humans. The O-antigen provides a protective mechanism also exploited in other polysaccharide-based bacterial vaccines^5,6^ and has been since many years a promising target for vaccine exploration in *E. coli*.

A four-valent (ExPEC4V)^7^ and nine-valent (ExPEC9V)^8^ bioconjugate vaccine containing four O-antigen polysaccharides (O-serotypes O1, O2, O6 and O25) and nine O-antigen polysaccharides (O-serotypes O1, O2, O4, O6, O15, O16, O18, O25 and O75), respectively, are assessed in clinical studies towards development of a vaccine for prevention of IED.^9^

To estimate the O-serotype distribution of extra-intestinal pathogenic *E. coli* in patients with IED aged ≥60 years, a multicentre, prospective, observational study EXPECT-2 (NCT04117113) was conducted. Clinical results within this study reported by Doua *et al.*^10^ found that out of patients with IED aged ≥60 years in eight participating hospitals from seven countries, 80.4% had bacteraemic IED and half of infections was community-acquired, with the urinary tract the most common source of infection. Sepsis and septic shock were reported in 72.1% and 10.0% of patients, respectively. Kidney dysfunction was the most common complication (12.9%) and overall in-hospital mortality was 4.6%.

This study is part of the EXPECT-2 research project, complementing the clinical results reported by Doua *et al*.^10^ with a microbiological analysis of the available isolates from 238 patients with IED. The major objective was prediction of the theoretical coverage of vaccine candidates ExPEC4V and ExPEC9V based on *E. coli* isolates obtained from hospitalized patients aged ≥60 years with a diagnosis of IED. The O-serotype, ST, AMR profile and genomic diversity of the isolates were obtained.

## Methods

### Ethics

This study was conducted in accordance with the Declaration of Helsinki, Good Clinical Practices and applicable regulatory requirements. In countries where no waiver was obtained, participants or their legally acceptable representatives provided their written consent to participate in the study after having been informed about the nature/purpose of the study, and the participation and termination conditions. The protocol, Informed Consent Form, and other relevant documents were approved by an Institutional Review Board/Independent Ethics Committee (IRB/IEC) before the study was initiated.

### Trial design and patient inclusion

EXPECT-2 (NCT04117113) was a prospective, observational, multicentre, hospital-based study conducted in eight participating hospitals, including two sites in Japan and one site each in the USA, Canada, France, Germany, Italy and Spain. The study took place between October 2019 and January 2021. The diagnosis of IED was determined by the microbiological confirmation of *E. coli* from blood, urine or an otherwise sterile body site in the presence of requisite criteria of systemic inflammatory response syndrome (SIRS), SOFA or quick SOFA (qSOFA).^1^

Patients were eligible for data collection if they were aged ≥60 years and hospitalized with a clinical diagnosis of IED. For countries where no waiver for informed consent had been obtained before data collection, a signed participation agreement/ICF/IAF allowing data collection and source data verification was obtained in accordance with local requirements.

All isolates from this *E. coli* dataset were stored in microbanks at −80°C at the local sites until shipment to the central laboratory at the University of Antwerp.

### Bacterial identification

Control of viability of the stored isolates was performed by culture at the central laboratory. Identification at species level was confirmed by MALDI-TOF MS (Bruker Daltonics, Bremen, Germany).

### Antimicrobial susceptibility testing (AST)

The MIC of 24 antimicrobial agents (Table S2, available as Supplementary data at *JAC* Online), covering 10 different drug classes, were determined for the full dataset by microdilution method using a commercial panel (Sensititre Gram-negative GN6F AST plate, ThermoFisher Scientific). Reading of MIC values was performed with a semi-automated digital MIC viewing system (Vizion^™^, ThermoFisher) and results were interpreted according to the 2023 (v.13.0) EUCAST criteria.^11^ MDR was defined as non-susceptibility to at least one agent in three or more antimicrobial categories.^12^

### Determination of O-serotypes

The O-serotype was determined as a combination of *in silico* O-genotyping and agglutination O-serotyping. O-genotyping was conducted based on WGS with identification of unique O-serotype specific sequences of *wzy*, *wzx*, *wzt* and *wzm* genes following guidelines and using reference sequences^13^ and were determined with the O-serotyper v.0.1 (Janssen Vaccines & Prevention, Leiden, the Netherlands).^14^ In case of negative O-genotyping results or in instances where the *in silico* result corresponded to an O-serotype belonging to one of the nine following serotypes included in the EXPEC9V vaccine candidate: O1, O2, O4, O6, O15, O16, O18, O25, O75, an O-serotyping agglutination assay (SSI Diagnostica, Hillerød, Denmark) was performed to confirm the O-genotyping result.

### Whole-genome sequencing

For Illumina short-read sequencing, genomic DNA was isolated from all isolates using the Lucigen MasterPure Complete DNA and RNA Purification kit, according to the manufacturer’s protocol. Sample and library preparation was done using Nextera XT kits (Illumina Inc, San Diego, CA, USA). Libraries per isolate were pooled and adjusted to equal molar quantities. Sequencing was done using MiSeq v.2, 500 cycle, PE 2 × 250 bp (Illumina Inc., USA). Raw sequencing data were quality-assessed using FastQC, cleaned using Trimmomatic v.0.4.2 with default parameters for adapter removal and quality trimming. Contamination (<5%) and completeness (>95%) were confirmed with CheckM v.1.1.2 (taxonomy_wf species ‘*Escherichia coli*’).

For PacBio long-read sequencing, genomic DNA was isolated from two isolates collected from the same patient (EXP200133 and EXP200135) and selected for long-read sequencing due to major differences in AMR genes and AST, using the Qiagen^®^ MagAttract^®^ HMW kit (Qiagen) according to the manufacturer’s protocol. Isolated DNA was sheared using Covaris G-tubes to obtain fragments with size distributions around 8–12 kb. Barcoded SMRT bell^™^ libraries were prepared with the SMRTbell Template kit. Sequencing was performed using the PacBio Sequel (Pacific Biosciences, CA, USA) with 2 h pre-extension and 10 h movie time in V3 SMRTCell (1M). PacBio sequencing data were processed, demultiplexed and assembled using SMRTLink v.10.2.

### Assembly, annotation and resistome analysis

Generation of the assemblies, annotations and resistomes was done using an in-house developed bacterial WGS pipeline, BacPipe v.1.2.6.^15^ Using this pipeline, *de novo* genome assemblies were made using SPAdes v.3.13.0 and genome assembly quality including number of contigs, total length and N50, was checked by QUAST v.5.0.2. Minimal average full depth coverage needed to be 50 times. Assemblies were annotated using Prokka v.1.11.1. The resistome was determined using the ResFinder v.2.1, CARD v.5.1 and ResFams v.1.2 databases with default parameters (90% identity and 60% coverage). Mutations in genes conferring resistance to fluoroquinolones (*gyrA* and *parC*) were identified with SRST2 v.0.2.0.^16^ SNP variants between paired isolates were detected with CLC genomics workbench v.9.5.3.

### Availability of WGS data

All sequence data from this study have been deposited under BioProject ID PRJNA1021534.

### MLST typing, identification of phylogroups and fimbria typing

*In silico* MLST based on seven conserved housekeeping genes and assignment to allelic numbers and STs was performed using the Achtman MLST locus/sequence definitions database.^17^*In silico* ClermonTyping method and its associated web-interface, the ClermonTyper v.1.4.0 was used for *E. coli* phylogroup determination.^18,19^*In silico* Fimbria Typing was done using FimTyper v.1.1.^20^

### Phylogenetic analysis

Parsnp v.1.7.4^21^ was used to create a core genome alignment of the nucleotide sequences from the unique isolate collection. SNPs within the alignment were filtered with Gubbins v.2.4.1^22^ and used to generate a phylogenetic tree with RAxML v.8.2.12,^23^ using the GTRGAMMA nucleotide substitution model. To obtain statistical support for the phylogeny, 1000 bootstraps were performed. The phylogenetic tree and isolate metadata were visualized with iTOL.^24^

## Results

### IED patients and collection of E. coli isolates

**Figure 1.**
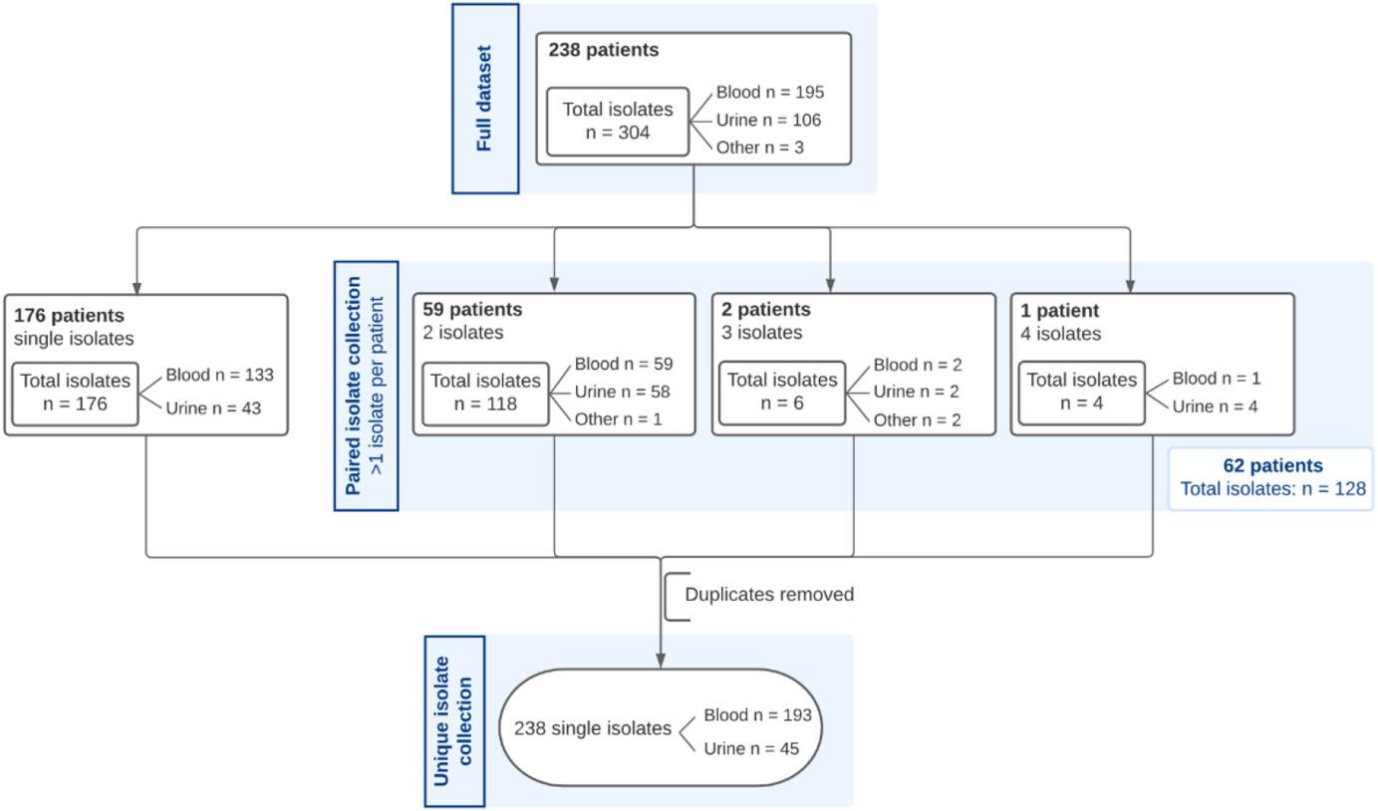
Overview of isolate collections used for analyses. The full data collection contains 304 *E. coli* isolates. The unique isolate collection contains 238 isolates whereas the paired isolate collection comprises 128 isolates.

In the study, 304 *E. coli* isolates were included from a total of 238 unique IED patients, with 195 isolates from blood (64.1%), 106 from urine (35.0%) and three from other sterile body sites (0.9%) (Table S1). A subset of the isolates, comprising one representative *E. coli* isolate per IED patient, hereafter referred to as the *unique isolate collection*, included a total of 238 individual isolates with 193 isolated from blood and 45 from urine (Figure 1). For the dedicated analysis of paired isolates, another sub-selection was made consisting of multiple isolates per patient (*n* = 128 from 62 patients), hereafter called the paired isolate collection.

### E. coli genetic diversity and dominance of O25b-ST131

**Figure 2.**
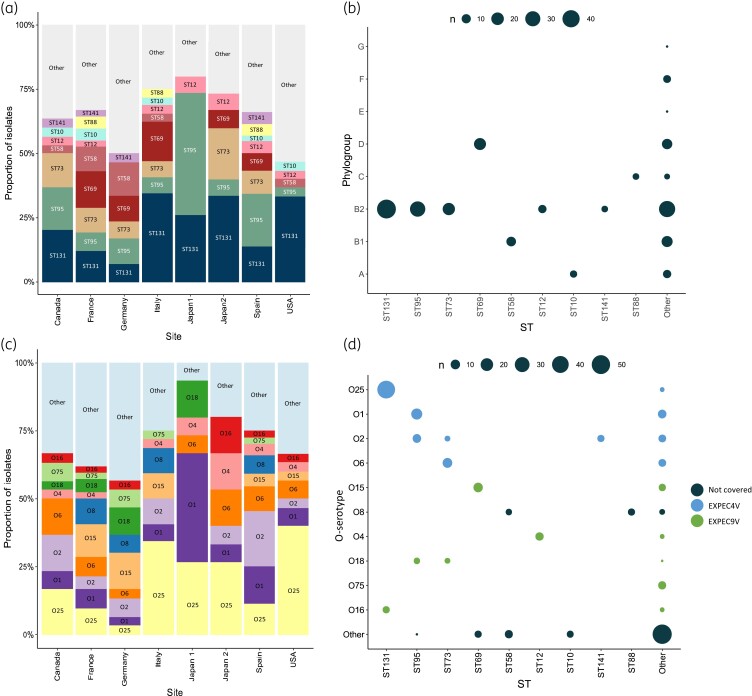
Distribution of ST (present in ≥5 isolates) and O-serotypes (present in ≥7 isolates). STs and O-serotypes present in, respectively, five or fewer and seven or fewer isolates are summarized as ‘other’. (a) The proportion of STs across hospital sites. (b) The pairwise correlation of STs with Clermont phylogroups. The magnitude of the circles indicates the number of isolates following the legend. (c) The proportion of O-serotypes across hospital sites. D The pairwise correlation of O-serotypes and ST. The magnitude of the circles indicates the number of isolates following the legend.

Among the unique isolate collection, genetic diversity was observed across and within study centres, resulting in the identification of 80 different STs (Figure 2a and Table S1). The nine most common STs (*n* = 152, 63.8%) were each found in at least five isolates and included ST131 (*n* = 49, 20.6%), ST95 (*n* = 31, 13%), ST73 (*n* = 19, 8%), ST69 (*n* = 18, 7.5%), ST58 (*n* = 11, 4.7%), ST12 (*n* = 8, 3.3%), ST10 (*n* = 6, 2.5%), ST88 (*n* = 5, 2.1%) and ST141 (*n* = 5, 2.1%). Seventy-one additional STs each occurred in less than five isolates from the unique isolate collection (*n* = 86, 36.1%). These STs were found in four isolates (one ST, *n* = 4, 1.7%), three isolates (four different STs, *n* = 12, 5%), two isolates (10 different STs, *n* = 20, 8.4%) or only one isolate (50 different STs, *n* = 50, 21%).

Most of the 238 unique isolates belonged to phylogroup B2 (*n* = 147, 62%). Other identified phylogroups were phylogroup D (*n* = 31, 13%), B1 (*n* = 26, 11%), A (*n* = 14, 6%), C (*n* = 9, 4%), F (*n* = 7, 3%), E (*n* = 2, 0.8%) and G (*n* = 2, 0.8%). *E. coli* isolates from the three most found ST types (ST131, ST95, ST73) all belonged to phylogroup B2 (Figure 2b).

Forty-seven O-serotypes were detected among the unique isolates, while for nine isolates (3.8%) no O-serotype could be determined (Table S1). The six most common O-serotypes, each represented by 10 or more isolates from the collection, were O25 (*n* = 46, 19.3%), O1 (*n* = 23, 9.7%), O2 (*n* = 22, 9.2%), O6 (*n* = 17, 7.1%), O15 (*n* = 15, 6.3%) and O8 (*n* = 12, 5%) (Figure 2c). These six O-serotypes were found in more than half of all isolates (*n* = 135, 56.7%). Twenty-four O-serotypes were each found in one isolate (*n* = 24, 10%) in the collection. O-serotypes mostly correlated with specific STs (Figure 2d), with the most common correlations between O25b-ST131 (*n* = 44, 18%), O6-ST73 (*n* = 13, 5%) and O1-ST95 (*n* = 16, 7%).

**Figure 3.**
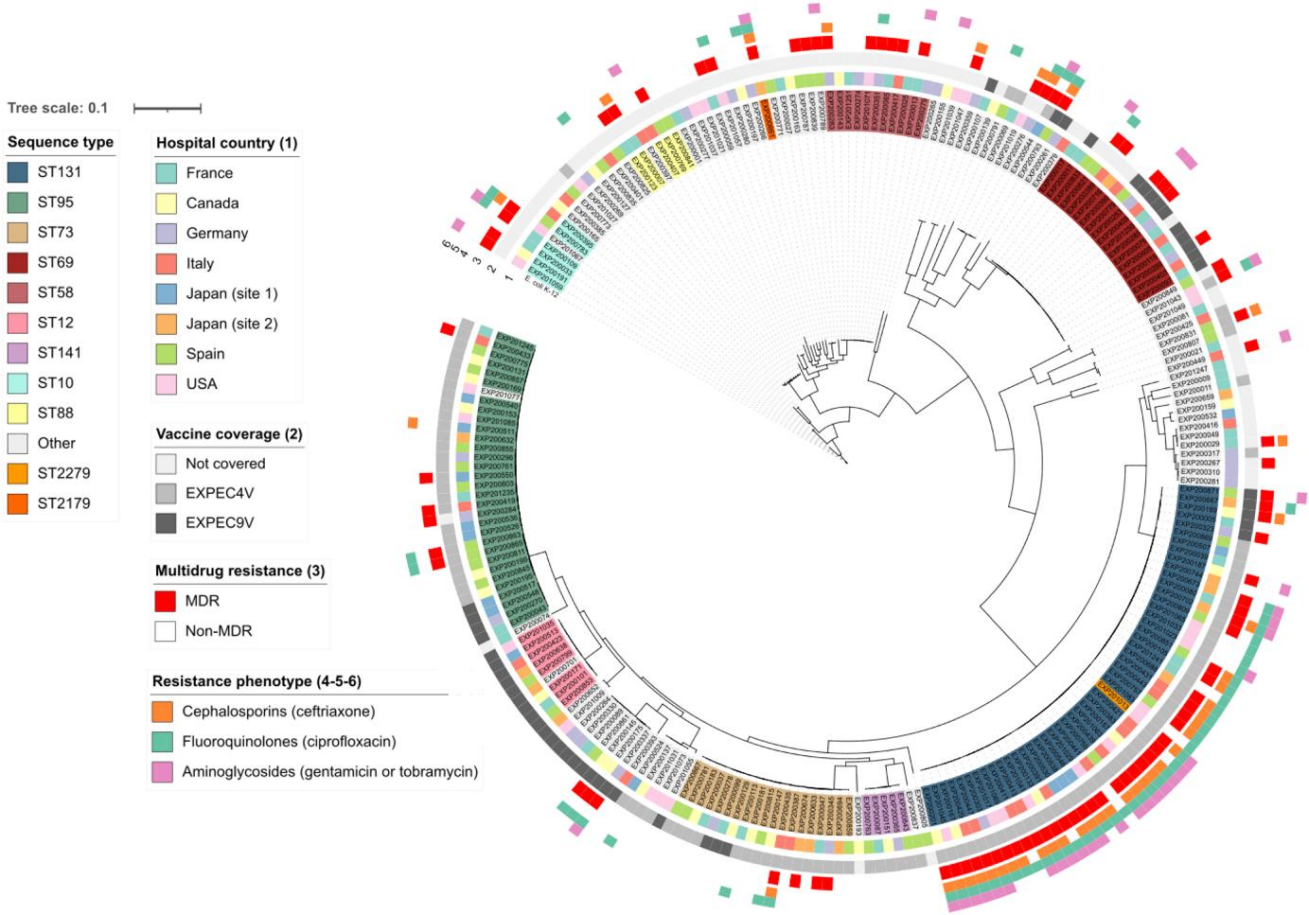
The population structure of extra-intestinal pathogenic *E. coli* is represented by a midpoint-rooted maximum-likelihood phylogenetic tree of the unique isolate collection (238 isolates), based on 1000 bootstraps. Core genomes of the isolates in this study and the reference genome *E. coli* K-12 were aligned. The tree is based on 139 932 SNPs. Coloured ranges represent predominant STs found in more than five isolates. Rings 1, 2 and 3 denote the hospital site, vaccine coverage by ExPEC4V and ExPEC9V, and multidrug resistance (MDR). Rings 4, 5 and 6 show resistance to cephalosporins, fluoroquinolones and aminoglycosides. The scale bar indicates the number of SNPs per site in the core genome alignment. The tree was visualized using iTOL.

The *E. coli* population is genetically diverse, with the different STs presenting separate branches across the overall phylogeny (Figure 3). The dominant STs were found to be globally dispersed and there is no country-specific signature of particular STs.

### Prevalence of antimicrobial resistance

Across the unique isolate collection, the highest resistance rates were reported for trimethoprim/sulfonamides (cotrimoxazole) and tetracyclines (31%, each). Substantial rates of resistance were also observed for fluoroquinolones (27.7%), third-generation cephalosporins (17.6%) and aminoglycosides (16.0%). No isolates were resistant to colistin or to carbapenems. At a geographic level, the highest proportion of resistance to third-generation cephalosporins, fluoroquinolones and aminoglycosides was observed in isolates from North America and Japan (Table 1). Eighty-nine isolates were characterized as MDR (37.4%), which were found at all eight participating sites (Table S2).

**Table 1.** Prevalence of antibiotic resistance following EUCAST breakpoints

Class of antibiotic	Europe (*n* = 148)	North America (*n* = 60)	Eastern Asia (*n* = 30)	Unique isolate collection (*n* = 238)
	Number of isolates (%)			
Susceptible	47 (31.8)	23 (38.3)	15 (50)	85 (35.7)
MDR^a^	54 (36.5)	23 (38.3)	11 (36.6)	89 (37.4)
Resistance to single class^b^				
Aminoglycosides (gentamicin/tobramycin)	18 (12.2)	14 (23.3)	6 (20)	38 (16.0)
β-lactam/β-lactamase inhibitor combination (piperacillin-tazobactam)	6 (4.1)	4 (6.7)	2 (6.7)	12 (5.0)
Extended-spectrum cephalosporin (ceftriaxone/ceftazidime)	19 (12.8)	16 (26.7)	7 (23.3)	42 (17.6)
Fluoroquinolones (ciprofloxacin/levofloxacin)	36 (24.3)	20 (33.3)	10 (33.3)	66 (27.7)
Folate synthesis inhibitor (trimethoprim/sulfamethoxazole)	52 (35.1)	15 (25.0)	7 (23.3)	74 (31.1)
Tetracyclines (tetracycline/minocycline)	52 (35.1)	16 (26.7)	6 (20.0)	74 (31.1)
Resistance to last resort antibiotics				
Colistin	0	0	0	0
Ertapenem	0	0	0	0
Imipenem	0	0	0	0
Meropenem	0	0	0	0
Ceftazidime/avibactam	0	0	0	0

^a^MDR: multidrug resistant defined as non-susceptibility to at least one agent in three of more antimicrobial categories.

^b^The degree of drug resistance was based on susceptibility to representative antibiotics in the following six classes of antimicrobial drugs: aminoglycosides, extended-spectrum penicillins (piperacillin-tazobactam) β-lactam/β- lactamase inhibitors, expanded-spectrum cephalosporins, folate synthesis inhibitor and tetracyclines.

Forty of the 89 MDR *E. coli* belong to ST131 (*n* = 39) or related ST2279 (*n* = 1, EXP201013). These isolates are also phylogenetically related and all part of the clonal complex (CC) 131 (Figure 3). One MDR isolate from a blood specimen of a patient in Japan belonged to ST2179 (EXP200661), phylogroup B1 and was phylogenetically unrelated to the other MDR isolates. It carried a *bla_CTX-M-65_* gene (an allelic variant belonging to CTX-M group 9) as well as a large variety of other AMR genes (16 genes, nine classes of antimicrobial agents). The ST2179 lineage has been recently reported from *E. coli* recovered from horses in Brazil,^25^ retail meat in Portugal as well as pigs and pet animals in Switzerland. While CTX-M-65-producing ST2179 *E. coli* had already been reported to occur in humans as commensal organisms, to our knowledge it had not yet been reported in the setting of an invasive bacterial infection in humans.

With regards to ESBL genes, apart from this particular ST2179 strain, all but one *E. coli* isolates of ST131/ST2279 carried a *bla_CTX-M-15_* gene in association with *bla_OXA-1_*, *aac(6’)-Ib-cr*, *catB3* as well as three mutations in the QRDR domain of *gyrA*/*parC* (GyrA S83L, GyrA D87N and ParC S80I) that are typically known to be associated with resistance to fluoroquinolones in *E. coli*. One single ST131 isolate harboured *bla_CTX-M-14_*.

### Association between O-serotypes, AMR and vaccine coverage

**Figure 4.**
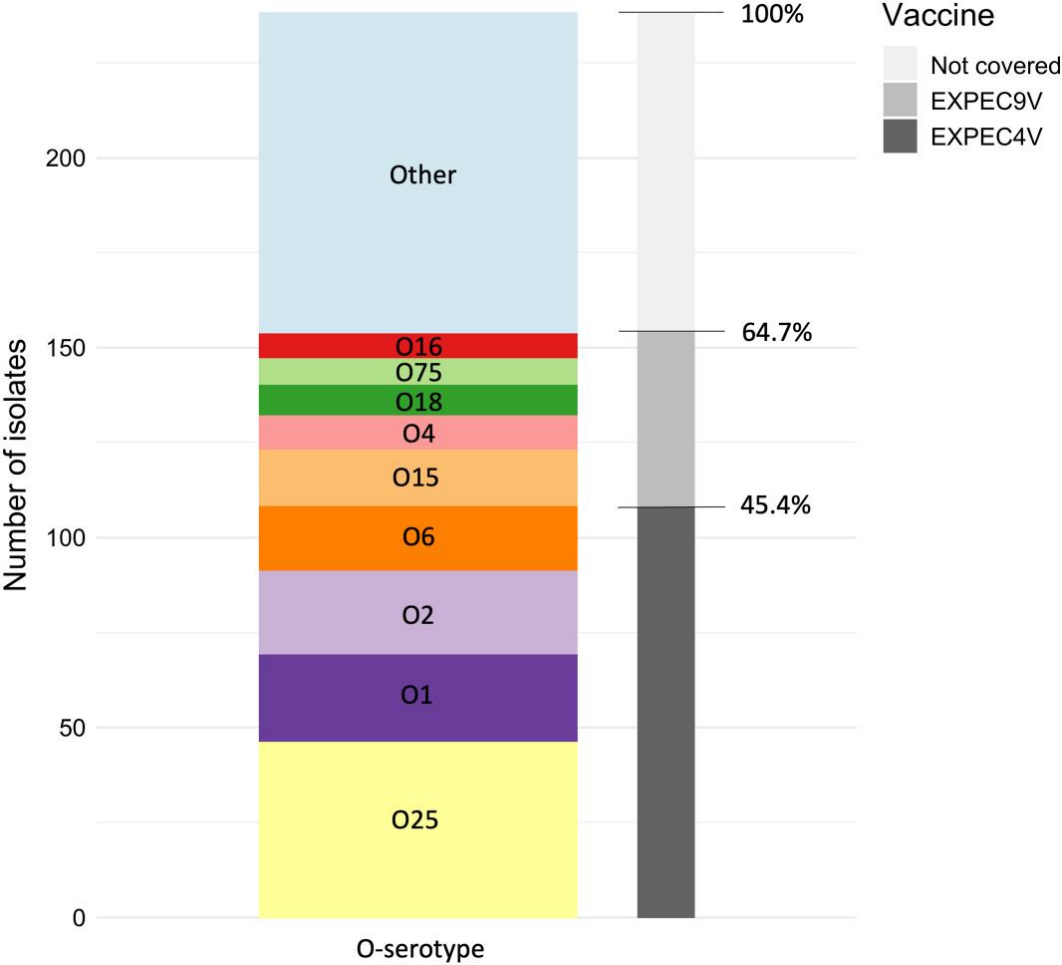
Number and ratio of isolates theoretically covered by the ExPEC4V and ExPEC9V vaccine candidate. The bar chart is coloured by the prevalence of O-serotypes in this study collection (unique isolate collection). The coverage in the ExPEC4V and ExPEC9V vaccines is coloured in shades of grey.

From the 238 unique isolates, 108 (45.4%) were found to carry serotypes that are included in the ExPEC4V vaccine candidate (Figure 4), whereas 154 out of 238 isolates (64.7%) contained O-antigens covered by the ExPEC9V vaccine. The most frequently found O-serotypes that were not covered by either of the vaccines were O8 (*n* = 12), members of the O77 group (i.e. O17/O44/O73/O77/O106, *n* = 6), O13 (*n* = 5), O9 (*n* = 5) and O107/O117 (*n* = 4).

Out of the 89 *E. coli* isolates that displayed a MDR pattern, 48 (53.9%) were covered by EXPEC4V and 59 (66.3%) were covered by EXPEC9V. By far the most prevalent serotype associated with MDR *E. coli* was O25b always in association with ST131 that accounted for 40% of all MDR isolates (Tables S4 and S5).

Besides O25b, serotype O1 (*n* = 5; two in ST95, one each in ST59, ST648 and ST1177), O2 (*n* = 4; three in ST95, one in ST73), O15 (*n* = 5; four in ST69, one in ST393), O18 (*n* = 1; in ST1193) and O75 (*n* = 2; in ST1193) have all been reported previously in association with MDR *E. coli* isolates from human infections.^4^ Of note, however, 13 of the 29 (44.8%) MDR *E. coli* isolates carrying non-vaccine serotypes belong to well-established or emerging MDR lineages of *E. coli* (ST58 (*n* = 5), ST10/ST744 (*n* = 2), ST38/ST963 (*n* = 2), ST117, ST357, ST973, ST2179 (*n* = 1 each) that have been reported from animals, farm environment or food products, human intestinal carriage samples and clinical specimens from human infections.^4,26,27^

### Analysis of paired isolates collected from blood and urine

From 62 patients, more than one isolate (*n* = 128/304) was collected, which we jointly called the paired isolate collection. Most of these isolates (113 isolates from 55 patients) harbour identical ST, O-serotype, AMR and AST (Table S3). For 37 isolate pairs (59.7%), SNP diversity was limited to ≤10 SNPs. Within-host diversity exceeded 10 SNPs in 25 pairs. One patient carried two O25b-ST131 isolates (EXP200133 and EXP200135) originating from a urine and blood sample, collected on the same day. In the blood isolate, six resistance genes *(aac(3)-IIa*, *bla_OXA-1_*, *bla_CTX-M-15_*, *catB3*, *dfrA17*, *aac(6’)-Ib-cr*) were detected that were not found in the urine isolate. The blood isolate also showed phenotypic resistance to nine additional antibiotics (ampicillin, ampicillin-sulbactam, aztreonam, cefazolin, cefepime, ceftazidime, ceftriaxone, gentamicin, tobramycin) as compared to the urine isolate. Long-read sequencing of both isolates from this patient revealed a plasmid carrying these resistance genes in-between transposases (Tn3 family transposase and IS6 family transposase). The full plasmid sequence (GenBank accession no. OR611935) was resolved and found to be 116 336 bp in length (Figure S1).

## Discussion

Globally, the increasing burden of invasive disease due to *E. coli* and the continued emergence of AMR among *E. coli* strains is of great concern. There is a knowledge gap regarding the global distribution of *E. coli* ST and O-serotypes associated with IED (including sepsis and bacteraemia), with the exception of two specific studies in the UK^28^ and France^29^ describing local findings focused on bacteraemia, as well as a collection of retrospective surveillance studies describing global O-serotype epidemiology between 2011 and 2017^30^ and genomic data from human commensal *E. coli* in France between 1980 and 2010.^31^ We present a recent, global prospective study on *E. coli* isolates from hospitalized IED patients combining O-typing results with phylogeny, and with a detailed comparison of AMR genotypes as determined with WGS and phenotypes with AST.

*E. coli* strains belonging to O25b-ST131 present a growing challenge for clinicians due to their frequent association with MDR, their increasing prevalence worldwide and ability to cause localized outbreaks of IED.^30^ The proportion of MDR isolates of 37% in our study of which 40% are O25, is comparable to the one reported in Oxfordshire, UK, where 44% (1434 of 3278) of the invasive *E. coli* isolates were found to be MDR. However, this proportion is much higher than reported by Weerdenburg *et al.*^30^ in a larger global study (with more than 3200 invasive *E. coli*), where approximately 10% of the isolates were classified as MDR and 60% of MDR isolates were O25. These differences might reflect regional differences, variability in MDR definition and/or antibiotic classes that were tested.

Epidemiological data about extra-intestinal *E. coli* in sub-Saharan Africa is still relatively scarce, but studies in Malawi,^32,33^ Tanzania,^34^ Uganda,^35^ South Africa^36^ and the Democratic Republic of Congo^37^ show a highly diverse *E. coli* population ST131 being the predominant ST.

The increasing levels of AMR among Gram-negative bacteria, including *E. coli*, is a global threat and is one of the main drivers for prophylactic approaches such as vaccine development. A nine-valent vaccine (EXPEC9V) for the prevention of IED is currently being tested in a pivotal vaccine efficacy trial (E.mbrace study; ClinicalTrials.gov Identifier NCT04899336). Our data support that ExPEC9V, as well as the earlier tested EXPEC4V, candidate vaccines cover a substantial number of the *E. coli* strains collected in this study, with a predicted vaccine coverage of approximately 45% for EXPEC4V and 65% for EXPEC9V. This finding is in line with previous findings from patients of all ages in the UK (46% and 72%; 2008–2018)^28^ and results from patients aged >60 years across Europe, North America, Asia-Pacific and South America (47% and 68%; 2011–2017).^30^ In commensal *E. coli* isolates from healthy individuals in France, the four O-serotypes covered by EXPEC4V represented nearly one-quarter (24%; 1980–2010) of the isolate collection.^31^ Previous implementation of the serotype-based pneumococcal vaccine led to an increased incidence of the serotypes that were not targeted by the vaccine.^38,39^ Pneumococcal vaccines induce immunity that impacts pneumococcal nasopharyngeal carriage, thereby applying a selective pressure to switch serotypes to escape the immune response. Unlike *Streptococcus pneumoniae*, *E. coli* normally inhabits the intestinal tract, representing a distinct immunological compartment. It remains unknown whether ExPEC4V and ExPEC9V apply the same selective pressure in the gut as pneumococcal vaccines do in the nasopharynx. Stool sample analysis including O-serotype prevalence and general metagenomic profiling revealed no indication of vaccine impact on *E. coli* prevalence, countering the notion of vaccine-induced serotype replacement.^40^ Close monitoring via surveillance and additional microbiome studies will be crucial to assess the risk of serotype replacement. Altogether, our findings support the further development and theoretical coverage of O-antigen based vaccines targeting *E. coli*. However, ongoing and future studies will need to provide evidence whether such vaccines show protective efficacy against the vaccine serotypes and assess the cost-benefit ratio of a vaccine-based prophylactic approach.

One study strength is that in a subset of patients more than one *E. coli* isolate was obtained from different body sites, allowing for a comparison of paired isolates from the same patient and thus assessment of the within-host diversity of the *E. coli* isolates both at the genomic and phenotypic levels. These data allowed us to confirm that in most of the cases the collected *E. coli* strains from blood and urine were genetically identical. However, in a small number of cases (four patients) different strains were observed, indicating a co-infection of multiple strains and highlighting the potential impact of within-host diversity. Alternatively, faecal contamination of the urine sample could lead to detection of an isolate that was not responsible for IED. One blood isolate harboured a plasmid with six resistance genes. Loss of this plasmid in the urine isolate compared to the blood isolate could have occurred during re-isolation and/or storage of the isolates.

Our study is part of the EXPECT-2 research project (ClinicalTrials.gov #NCT04117113) and complements the paper by Doua *et al.*^10^ in which the clinical presentation, characterization of IED by infection acquisition setting and outcome was reported in detail. Overall, the proportion of patients with community-acquired (50%) and healthcare-associated (30%) IED reflects the proportion reported in other similar studies carried out in elderly adult patients in different countries and continents.^41,42^

One of the limitations of our study is the potential bias of this collection by including only one enrolling site per country, except for Japan, which might not be representative of the distribution of *E. coli* isolates at the country level. In addition, the participating centres used their own criteria and protocols for obtaining blood culture collection, sample transport and storage of isolates. Further, a selection bias at the patient level cannot be excluded as the proportion of community- versus healthcare-acquired infection, comorbidities and severity of infection at time of enrolment could possibly have varied at the different sites. Moreover, this study was a prospective study of relatively short duration (between 2019 and 2021). This may not enable us to capture the possible emergence of new clonal lineages involved in IED or population genetic structure evolution within STs that may only appear in longitudinal surveys over longer periods (10–20 years).^43–45^

In conclusion, the *E. coli* isolates causing IED are diverse, with the most frequent being O25b-ST131, a strain with high rates of MDR. Our data showing the distribution of the phylogeny, STs, O-serotypes and AMR profiles of *E. coli* isolates may provide additional epidemiological and microbiological information and inform the development of multivalent conjugate vaccines that target *E. coli* O-antigens.

## Supplementary Material

dkae182_Supplementary_Data

## Transparency declarations

J.D.: Employee and stock option owner of Janssen Research & Development, a pharmaceutical company of Johnson & Johnson. B.M.: Employee and stock (option) owner of Janssen Research & Development, a pharmaceutical company of Johnson & Johnson. J.G.: Employee and stock (option) owner of Janssen Vaccines & Prevention, a pharmaceutical company of Johnson & Johnson. O.G.: Employee of Janssen Research & Development, a pharmaceutical company of Johnson & Johnson and a shareholder of Johnson & Johnson. B.S.: Employee and share owner of Johnson & Johnson. M.S.: Employee and unrestricted share owner of Janssen Vaccines, Branch of Cilag GmbH International, a Johnson & Johnson company. J.P.: Employee and stock option owner of Janssen Research & Development, a pharmaceutical company of Johnson & Johnson. M.B.: Grants paid to University Medical Center Utrecht (UMCU) from CureVac, Janssen Vaccines, Merck and Novartis; honoraria payments to MJMB from Takeda; participation on a Data Safety Monitoring Board supported by AstraZeneca, Merck, Novartis, Pfizer, Sanofi and Spherecydes. All other authors: Nothing to declare. Author(s) J.D., O.G., J.G., B.S., M.S. and J.P. belong to EFPIA (European Federation of Pharmaceutical Industries and Association) member companies in the Innovative Medicines Initiative Joint Undertaking (IMI JU), and costs related to their part in the Development were carried by the respective company as in kind contribution under the IMI JU scheme.

## Supplementary data

Figure S1 and Tables S1 to S5 are available as Supplementary data at *JAC* Online.
