# Supplementary material for: A microbiological and genomic perspective of globally collected Escherichia coli from adults hospitalized with invasive E. coli disease
Source: J Antimicrob Chemother. 2024 Jul 13;79(9):2142–51. doi: 10.1093/jac/dkae182 (PMC11368426; doi:10.1093/jac/dkae182)
Supplement: dkae182_Supplementary_Data [file dkae182_supplementary_data.zip › SupplementaryTables_4-5.docx]

**Supplementary Table 4.** Most prevalent O-serotypes and drug resistance

N (%) is the number (percentage) of isolates that were susceptible or resistant. Cumulative % refers to the cumulative percentage of isolates that were susceptible or resistant with addition of each percentage by individual O-genotype/serotype.

The list includes the list of the O serotypes (or groups of serotypes) by decreasing order of frequency for the 15 O serotypes with a prevalence > 1% in the total study population.

*MDR : is resistant to at least 1 agent in three or more than 3 classes of antimicrobial agents.

Other**: include any other less common O-genotypes/serotypes as well as isolates with no determined O-genotypes and/or not typeable by slide agglutination

|  |  |  |  |  |  |  |  |  |  |  |  |  | |  |  |
| --- | --- | --- | --- | --- | --- | --- | --- | --- | --- | --- | --- | --- | --- | --- | --- |
| O-serotype | | | Total (n=238) | |  | Susceptible (n=85) | |  | Resistant (<3 classes) (n=64) | |  | MDR* (n=89) | |  |  |
|  | | | n (%) | cumulative % |  | n (%) | cumulative % |  | n (%) | cumulative % |  | n (%) | cumulative % |  |  |
| O25 | | | 46 (19.3) | 19.3 |  | 3 (3.5) | 3.5 |  | 7 (10.9) | 10.9 |  | 36 (40.4) | 40.4 |  |  |
| O1 | | | 23 (9.7) | 29.0 |  | 10 (11.8) | 15.3 |  | 8 (12.5) | 23.4 |  | 5 (5.6) | 46.0 |  |  |
| O2 | | | 22 (9.2) | 38.2 |  | 11 (12.9) | 28.2 |  | 7 (10.9) | 34.3 |  | 5 (5.6) | 51.6 |  |  |
| O6 | | | 17 (7.1) | 45.3 |  | 8 (9.4) | 37.6 |  | 6 (9.4) | 43.7 |  | 3 (3.4) | 55.0 |  |  |
| O15 | | | 15 (6.3) | 51.6 |  | 2 (2.4) | 40.0 |  | 8 (12.5) | 56.2 |  | 5 (5.6) | 60.6 |  |  |
| O4 | | | 9 (3.8) | 55.4 |  | 6 (7.1) | 47.1 |  | 3 (4.7) | 60.9 |  | 0 (0.0) | 60.6 |  |  |
| O18 | | | 8 (3.3) | 58.7 |  | 5 (5.9) | 53.0 |  | 2 (3.1) | 64.0 |  | 1 (1.2) | 61.8 |  |  |
| O16 | | | 7 (3.0) | 61.7 |  | 2 (2.4) | 55.4 |  | 1 (1.6) | 65.6 |  | 4 (4.5) | 66.3 |  |  |
| O75 | | | 7 (3.0) | 64.7 |  | 4 (4.7) | 60.1 |  | 1 (1.6) | 67.2 |  | 2 (2.2) | 68.5 |  |  |
| O8 | | | 12 (5.0) | 69.7 |  | 1 (1.2) | 61.3 |  | 5 (7.8) | 75.0 |  | 6 (6.7) | 75.2 |  |  |
| O17/O44/O73/O77/O106 | | | 6 (2.6) | 72.3 |  | 2 (2.4) | 63.7 |  | 3 (4.7) | 79.7 |  | 1 (1.2) | 76.4 |  |  |
| O9 | | | 5 (2.2) | 74.5 |  | 0 (0.0) | 73.1 |  | 2 (3.1) | 82.8 |  | 3 (3.4) | 79.8 |  |  |
| O13 | | | 5 (2.2) | 76.7 |  | 2 (2.4) | 75.5 |  | 2 (3.1) | 85.9 |  | 1 (1.2) | 81.0 |  |  |
| O107/O117 | | | 4 (1.7) | 78.3 |  | 2 (2.4) | 77.9 |  | 0 (0.0) | 85.9 |  | 0 (0.0) | 81.0 |  |  |
| O101/0162 | | | 2 (0.8) | 79.1 |  | 0 (0.0) | 77.9 |  | 0 (0.0) | 85.9 |  | 2 (2.3) | 83.3 |  |  |
| Other** | | | 50 (21.0) | 100.0 |  | 18 (22.1) | 100.0 |  | 9 (14.1) | 100.0 |  | 15 (16.8) | 100.0 |  |  |

**Supplementary Table 5.** Drug resistance by antibiotic class in each O-serotype

| O-serotypes  n  (%) | Total  (N=238) | O25  (N=46) | O1  (N=23) | O2  (N=22) | O6  (N=17) | O15  (N=15) | O4  (N=9) | O18  (N=8) | O16  (N=7) | O75  (N=7) | O8  (N=12) | O17/ O44  (N=6) | O9  (N=5) | O13  (N=5) | O107/ O117  (N=4) | | O10/ O162  (N=2) | Other  (N=50) |
| --- | --- | --- | --- | --- | --- | --- | --- | --- | --- | --- | --- | --- | --- | --- | --- | --- | --- | --- |
| Susceptible | 85  (35.7) | 3  (6.5) | 10  (43.5) | 10  (45.5) | 8  (47.1) | 2  (13.3) | 6 (66.7) | 5 (62.5) | 2 (28.6) | 4  (57.1) | 1  (8.3) | 2 (33.3) | 0 | 2  (40) | 2  (50) | 0 | | 27  (54) |
| Aminoglycosides | 38  (16.0) | 25 (54.4) | 2  (8.7) | 0 | 0 | 2  (13.3) | 0 | 1  (12.5) | 1  (14.3) | 0 | 1  (8.3) | 0 | 1  (20) | 0 | 0 | 1  (50) | | 4  (8) |
| Cephalosporins | 42  (17.6) | 29  (63.1) | 2  (8.7) | 1  (4.5) | 0 | 1  (6.7) | 0 | 0 | 1  (14.3) | 0 | 2  (16.7) | 0 | 1  (20) | 1  (20) | 0 | 2  (100) | | 5  (10) |
| Fluoroquinolones | 66  (27.7) | 41  (89.1) | 3  (13.1) | 6  (27.2) | 0 | 1  (6.7) | 0 | 1  (12.5) | 2 (28.6) | 2 (28.6) | 3  (25) | 2 (33.3) | 2  (40) | 0 | 0 | 2  (100) | | 5  (10) |
| Penicillins | 140 (58.8) | 39  (84.8) | 13  (56.5) | 10  (45.5) | 9  (53) | 13  (86.7) | 3 (33.3) | 3  (37.5) | 5  (71.4) | 3 (42.9) | 9  (75) | 4 (66.7) | 5  (100) | 3  (60) | 2  (50) | 2  (100) | | 17  (34) |
| Trimethoprim/sulf | 76  (31.9) | 21  (45.7) | 4  (17.4) | 5  (22.7) | 4  (23.5) | 5  (33.3) | 0 | 1  (12.5) | 4 (57.2) | 3 (42.9) | 7  (58.4) | 1  (16.7) | 5  (100) | 1  (20) | 2  (50) | 2  (100) | | 102  (24) |
| Tetracyclines | 74  (31.1) | 21  (45.7) | 4  (17.4) | 4  (18.2) | 4  (23.5) | 10  (66.7) | 1  (11.1) | 1  (12.5) | 4 (57.2) | 1  (14.3) | 60  (50) | 0 | 1  (20) | 0 | 1  (25) | 2  (100) | | 16  (21.6) |

**
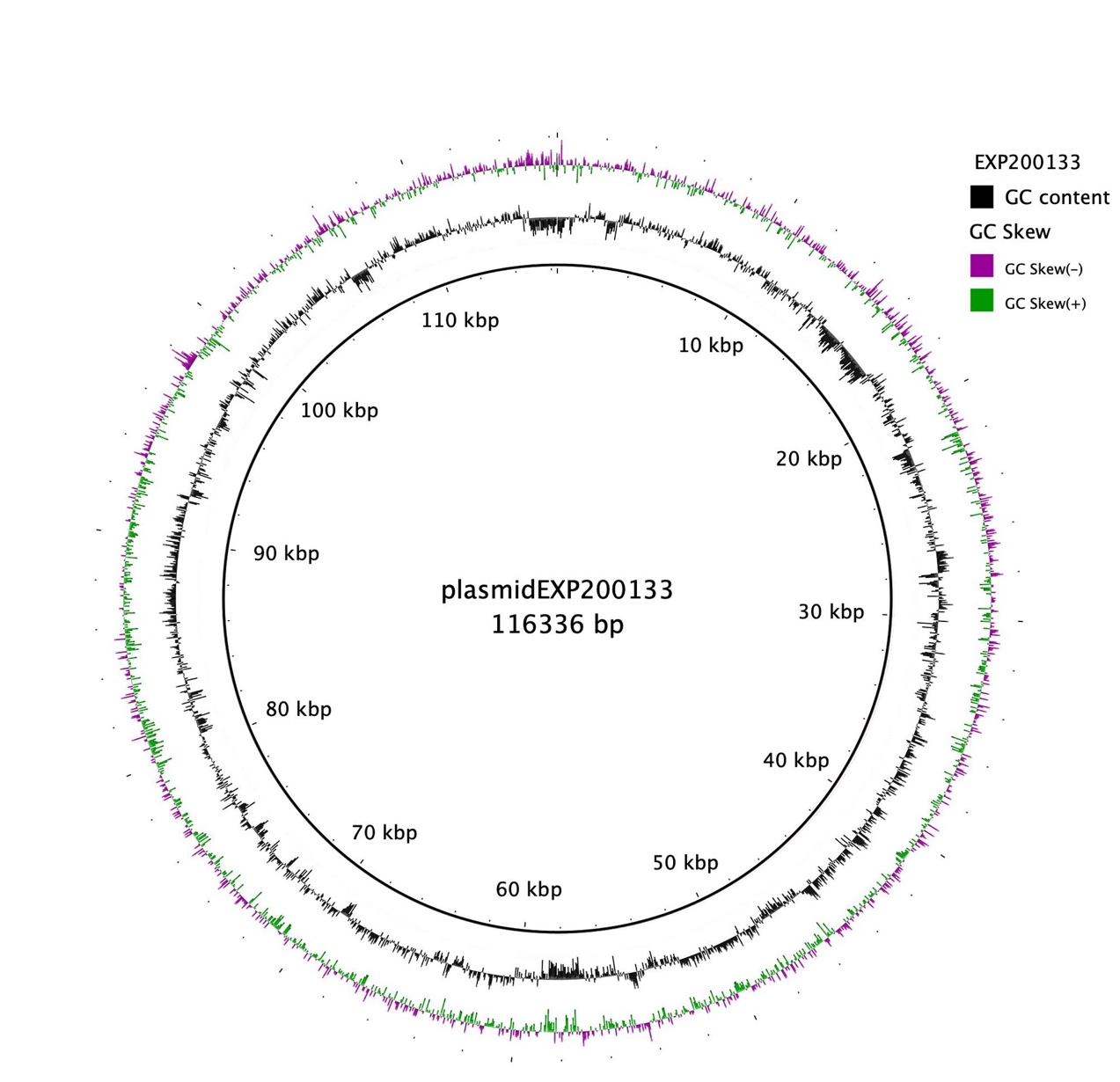
**

**Supplementary figure 1.** BRIG visualization of a plasmid in isolate EXP200133 encoding the genes *aac(6’)-Ib-cr*, *blaOXA-1*, *catB3* and *blaCTX-M-15,* located closely together and in-between transposases (Tn3 family transposase and IS6 family transposase).
